# Antimicrobial resistance pattern due to oxygen gap at high altitudes

**DOI:** 10.3389/fcimb.2025.1673102

**Published:** 2025-10-22

**Authors:** Zong Chao Yang, Zhang Rui Han, Xiang Yang Li, Ya Bin Duan

**Affiliations:** ^1^ Department of Clinical Pharmacy, Qinghai University Affiliated Hospital, Xining, China; ^2^ College of Pharmacy, Qinghai University, Xining, China

**Keywords:** high-altitude hypoxia, antimicrobial resistance, antibiotics, efflux pump, mechanism

## Abstract

Over the past 10 years, microbial resistance has seriously threatened human life and health, and the treatment of multidrug-resistant bacteria remains a challenge for clinicians, pharmacists, and infectious disease physicians. Bacterial resistance is affected by a variety of factors, such as the environment, economy, and drug abuse. This review compared the differences in bacterial resistance rates between high- and low-altitude areas and explored the relevant mechanisms of bacterial resistance in the low-oxygen environment of plateaus, providing new clinical research ideas for curbing the occurrence of bacterial resistance.

## Introduction

Plateau environments are characterized by low oxygen, low atmospheric pressure, strong ultraviolet radiation, drought, and a large temperature difference between day and night, among which low oxygen is the main factor affecting human activities (Eide and Asplund, 2012; Witt and Huerta-Sánchez, 2019; Mazel, 2019). For every 100 m of altitude, the atmospheric pressure drops by approximately 0.67 kPa, the partial pressure of oxygen drops by 0.14 kPa, and the air temperature drops by 0.6°C; at 5,000 m, the partial pressure of oxygen drops to half of that at sea level (An, 2019). There are three main regions in the world with altitudes greater than 4,000 m: the Tibetan Plateau, the Andes Mountains of South America, and the Ethiopian Plateau. The Tibetan Plateau covers an area of approximately 2.5 million km^2^, making it the highest and most extensive plateau in the world (Gilbert-Kawai et al., 2014). Approximately 80 million people live in high-altitude areas in China, of which roughly 8–10 million live in areas higher than 3,000 m above sea level (masl) (Zhu, 2020). With the current economy, a large number of people in the plain area enter high-altitude areas for work, sports training, tourism, business, and plateau border garrison activities. When the human body quickly enters plateau environments, the body undergoes a series of physiological and pathological changes to adapt to this hypoxic environment, which even causes diseases such as plateau pulmonary edema and plateau cerebral edema (Wu, 2006; Chen and An, 2012). Plateau environments not only change the physiological functions of the body but also have a certain impact on the life processes of microorganisms, such as the relative abundance of intestinal microbiota in these environments (Hu et al., 2015; Liu et al., 2021; Bai et al., 2022).

Antimicrobial resistance is a critical issue in global and local healthcare. Previous studies have found that the activity and expression levels of MDR1, BCRP, and MRP2 in the host efflux pumps in a plateau hypoxic environment are significantly increased, which increases the efflux of drugs, leading to drug resistance (Duan et al., 2022). Efflux pump systems in bacteria are also among the key mechanisms for the development of drug resistance. According to the data from the China Antimicrobial Surveillance Network, the antimicrobial resistance rate in the plateau hypoxic environment is significantly different from that in the plain area, and there are obvious regional differences. As such, it is critical to study the mechanism of drug resistance change and the prevention and control measures in the plateau area. This article reviews the antimicrobial resistance rate in plateau and plain areas and the related mechanisms of antimicrobial resistance affected by plateau hypoxia.

## Microbial resistance in the plateau of China

Qinghai Province is located in the Qinghai–Tibet Plateau, with an average altitude of more than 3,000 m, and the area from 3,000 to 5,000 masl is 532,000 km^2^, accounting for 76.3% of the total area of the province. The area 5,000 masl is 54,000 km^2^, accounting for 7.8% of the province’s total area. Tibet is located in the southwest of the Qinghai–Tibet Plateau, with an average altitude of more than 4,000 m. The Himalayan high mountains, located in southern Tibet, are composed of several east–west mountain ranges with an average altitude of approximately 6,000 m. Mount Everest, located on the border of China and Nepal in Dingri County, Tibet, has the highest peak in the world, with an altitude of 8,848.86 m. Few studies have been conducted on the occurrence of bacterial resistance in Qinghai and Tibet, and in-depth molecular epidemiology and resistance mechanism studies are lacking.

Shi et al. (2016) analyzed bacterial resistance in the People’s Hospital of the Tibet Autonomous Region in 2015, and the detection rates of methicillin-resistant *Staphylococcus aureus* (MRSA) and methicillin-resistant coagulase-negative *Staphylococcus* (MRCNS) were 33.9% and 62.7%, respectively. No vancomycin-resistant *Enterococcus faecalis* (VREF) or *Enterococcus faecium* strains were detected. The top three isolates among gram-negative bacteria were *Escherichia coli* (31.2%), *Klebsiella* spp. (25.9%), and *Acinetobacter baumannii* (*AB*) (15.5%). The resistance rates of *E. coli* to cefotaxime and ciprofloxacin were 42.6% and 56.7%, respectively. The prevalence of imipenem resistance in *Klebsiella pneumoniae* was 1.6%. *Pseudomonas aeruginosa* (*PA*) was <30% resistant to carbapenems, piperacillin/tazobactam, cefepime, ceftazidime, aminoglycosides (amikacin and gentamicin), and quinolones (ciprofloxacin and levofloxacin). More than 65% of *AB* strains were resistant to carbapenems. Shi et al. (2020) compared the bacterial resistance of a tertiary hospital in Tibet and a tertiary hospital in Shanghai in 2017 and found that no imipenem-resistant *K. pneumoniae* was detected in Tibet, whereas the detection rate of a tertiary hospital in Shanghai was 30.03%. There was no statistically significant difference in the resistance rates between the two hospitals. There were some differences in the drug resistance of *PA* between the two hospitals. The detection rate of carbapenem-resistant *PA* (CRPA) was 17.86% in Tibet and 29.86% in Shanghai (*p* = 0.025). The detection rate of MRSA in Tibet was 57.51%, which was significantly higher than that in Shanghai (41.63%) (*p* < 0.001). Vancomycin-resistant *Enterococcus* (VRE) was not detected in Tibet, and the detection rate in Shanghai was 0.6%; however, the resistance rate of enterococci to ampicillin in Tibet was higher than that in Shanghai (*p* < 0.001).

Ci et al. (2021) analyzed the bacterial resistance situation in the People’s Hospital of the Tibet Autonomous Region in 2018, where the MRSA detection rate was 53%, the MRCNS detection rate was 83%, vancomycin-resistant *E. faecium* was not found, no staphylococci insensitive to vancomycin and teicoplanin were found, and *Enterobacter* susceptibility to carbapenems, cephamycins, minocyclines, and enzyme inhibitors was >80%. The drug resistance rates to cefotaxime and ceftriaxone were >50.0%, the resistance rate of *PA* to carbapenems was >10.0%, *AB* was >70.0% resistant to most antimicrobial drugs, and the resistance rate to imipenem was 74%. Yang et al. (2014) analyzed the drug resistance of 198 strains of *Mycobacterium tuberculosis* in Lhasa, Tibet, and they found 125 drug-resistant strains, with a total resistance rate of 63.1% (125/198), a first-time drug resistance rate of 55.1% (64/116), and a retreatment drug resistance rate of 75.4% (40/53). Multidrug-resistant tuberculosis (MDR-TB) and extensively drug-resistant tuberculosis (XDR-TB) are characterized by prolonged treatment duration, high incidence rates, and low cure rates, making China one of the countries with the highest global burden of tuberculosis. The drug resistance of tuberculosis in Lhasa, Tibet, is higher than that in other parts of China, which may be related to the small population density, scattered residence, inconvenient transportation, patients mostly self-medicating at home, single medication, insufficient dosage, self-discontinuation of medication, stopping from time to time, and poor treatment compliance. Huang and Ji (2012) studied the resistance of MRSA and *Staphylococcus epidermidis* in two hospitals in Qinghai from 2010 to 2011, with detection rates of 52.6% and 85.7%, respectively; *E. faecalis* and *E. faecium* were resistant to vancomycin and teicoplanin, and the resistance rate of *E. coli* and *Enterobacter* to most of the tested drugs was >40.0%, of which the resistance rate of *E. coli* to quinolones was approximately 55.6%, and the rate of extended-spectrum β-lactamase (ESBL) production was 35.3%. The resistance rate of non-fermenting bacteria to antimicrobial drugs was 20.0%–40.0%. Drug resistance in intensive care units is more serious than that in other hospital wards, and there are obvious differences in bacterial resistance in different provinces and cities. Zhao et al. (2020) analyzed the drug resistance of *AB* in Qinghai Province from 2016 to 2018 and found that it had a high resistance rate to aminoglycosides and cephalosporin antimicrobials (40–50%), whereas the resistance rates to meropenem and levofloxacin were 30%–40%. There was no correlation between the frequency of antimicrobial drugs with levofloxacin, ceftazidime, meropenem, and gentamicin and the resistance rate of *AB* (*p* > 0.05). However, when the frequency of antimicrobial administration was low, the resistance rate of *AB* was positively correlated with the frequency of antimicrobial drugs, and the occurrence of drug resistance was mainly due to the selective pressure of antimicrobial drugs.

**Figure 1:**
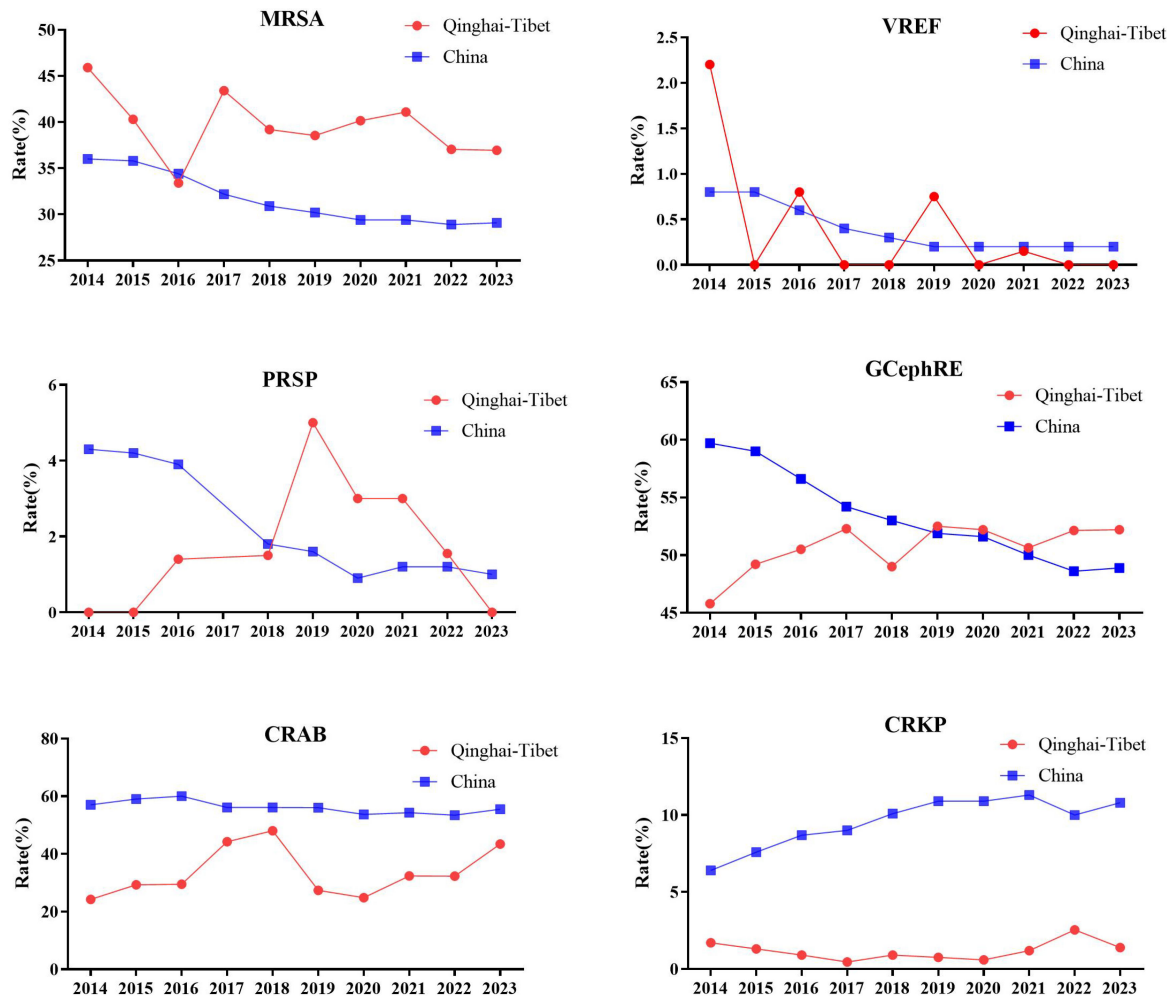
Line chart of drug resistance rate of MRSA, VREF, PRSP, GCephRE, CRAB, and CRKP in Qinghai–Tibet and China. From 2014 to 2023: Qinghai mean altitude above 3,000 m, and Tibet mean altitude above 4,000 m. MRSA, methicillin-resistant *Staphylococcus aureus*; VREF, vancomycin-resistant *Enterococcus faecalis*; PRSP, penicillin-resistant *Streptococcus pneumoniae*; GCephRE, third-generation cephalosporin Enterobacteriaceae; CRAB, carbapenem-resistant *Acinetobacter baumannii*; CRKP, carbapenem-resistant *Klebsiella pneumoniae*; Qinghai–Tibet: Qinghai and Tibet average drug resistance rate. China: The average drug resistance rate of Beijing (43.5 m), Henan (100 m), Liaoning (208 m), Hubei (20 m), Jiangsu (28 m), Jiangxi (217.7 m), Shaanxi (1,127 m), Shanxi (936 m), Anhui (119.3 m), Shanghai (4 m), Shandong (50 m), Chongqing (754 m), Hunan (365 m), Guangxi (397 m), Xinjiang (2,188 m), Inner Mongolia (1,000 m), Hebei (1,500 m), Yunnan (2,000 m), Guangdong (194 m), Fujian (298 m), Guizhou (1,100 m), Gansu (2,158 m), Hainan (120 m), Heilongjiang (244 m), Sichuan (1,056 m), Ningxia (1,000 m), Jilin (360 m), Zhejiang (186 m), Tianjin (3.3 m), Tibet (4,000 m), and Qinghai (3,000 m). Note: PRSP data missing in 2017.

Based on data from the China Antimicrobial Surveillance Network, we analyzed MRSA, VREF, penicillin-resistant *Streptococcus pneumoniae* (PRSP), third-generation cephalosporin-resistant Enterobacteriaceae (GCephRE), carbapenem-resistant *AB* (CRAB), and carbapenem-resistant *K. pneumoniae* (CRKP) detection rates between 2014 and 2023. There were significant regional differences in drug resistance among some bacteria. Considering Qinghai and Tibet as plateau areas above 3,000 masl, the detection rate of MRSA was significantly higher than the national average, and the detection rates of VREF, CRAB, and CRKP were significantly lower compared to the national average. In 2014, Qinghai Province had the highest VREF resistance rate (2.2%) and the lowest PRSP resistance rate (0%). GCephRE showed the lowest drug resistance rate (45.8%), followed by CRAB (24.2%). CRKP is also at a low national level of 1.7%. In 2020, Tibet had the highest MRSA detection rate in the country (46%). Both Qinghai and Tibet had the lowest VREF rate at 0%, whereas the prevalence of PRSP was the highest in Tibet (6%), and it was not detected in Qinghai. The detection rate of CRAB was the lowest in Qinghai and Tibet at 31.5% and 18.2%, respectively. Qinghai and Tibet also had the lowest CRKP detection rates in the country, at 1% and 0.2%, respectively. Between 2014 and 2023, the detection rate of MRSA in high-altitude areas was significantly higher than that in plain areas. Between 2014 and 2018, the detection rate of PRSP in the plateau area was significantly lower than the national average and increased significantly in 2019. Between 2014 and 2023, the detection rate of MRSA in high-altitude areas was significantly higher than that in plain areas. From 2014 to 2018, the detection rate of PRSP in the plateau area was significantly lower than the national average and increased significantly in 2019. From 2014 to 2019, the GCephRE detection rate in the plateau area was significantly lower than the national average, whereas the detection rates in 2020 were similar. Between 2014 and 2023, the detection rates of CRAB and CRKP in the plateau area were significantly lower than the national average (**Figure 1**). In summary, certain regional differences exist in bacterial resistance in China, and plateau hypoxic environments may affect the occurrence of bacterial resistance. For example, the detection rates of CRAB and CRKP in high-altitude areas were significantly lower than those in plain areas, indicating that plateau environments significantly affected the occurrence of antimicrobial resistance in *AB* and *K. pneumoniae*. In addition, the polygonal lines of the resistance rates of PRSP and MRSA coincide at some point in time, and bacterial resistance may increase or decrease sharply owing to the influence of various factors, such as the local economic level and medical conditions.

Between 2014 and 2023, there were significant differences in the detection rates of MRSA, PRSP, CRAB, and CRKP between the plateau and plain areas. According to the results of the bacterial resistance studies by various scholars, the drug resistance rates of CRAB, MRSA, CRPA, VRE, and CRKP were significantly different from those in the plain area (**Table 1**). In addition, there are significant regional differences in the drug resistance rates of *M. tuberculosis*. Many factors affect bacterial resistance, such as altitude, drug frequency, population density, and treatment compliance; therefore, the mechanism of the influence of a plateau hypoxic environment on bacterial resistance needs to be further clarified.

**Table 1:** Drug resistance rate of CRKP, CRAB, CRPA, MRSA, and VRE in Qinghai–Tibet and Shanghai.

Drug-resistant bacteria	Year	High altitude area (altitude)/drug resistance rate (%)	Low altitude area (altitude)/drug resistance rate (%)	References
CRKP	2009–2011	Tibet (4,000 m)/13%		(Du et al., 2013)
	2015	Tibet (4,000 m)/1.6%		(Shi et al., 2016)
	2017	Tibet (4,000 m)/0%	Shanghai (4 m)/30.03%	(Shi et al., 2020)
CRAB	2009–2011	Tibet (4,000 m)/19%		(Du et al., 2013)
	2015	Tibet (4,000 m)/68%		(Shi et al., 2016)
	2017	Tibet (4,000 m)/69.28%	Shanghai (4 m)/65.09%	(Shi et al., 2020)
	2018	Tibet (4,000 m)/74.7%		(Ci et al., 2021)
CRPA	2009–2011	Tibet (4,000 m)/20%		(Du et al., 2013)
	2015	Tibet (4,000 m)/22.6%		(Shi et al., 2016)
	2017	Tibet (4,000 m)/17.86%	Shanghai (4 m)/29.86%	(Shi et al., 2020)
	2018	Tibet (4,000 m)/12.5%		(Ci et al., 2021)
MRSA	2010	Qinghai (3,000 m)/52.6%		(Du et al., 2013)
	2017	Tibet (4,000 m)/57.51%	Shanghai (4 m)/41.63%	(Shi et al., 2020)
	2018	Tibet (4,000 m)/53%		(Ci et al., 2021)
VRE	2017	Tibet (4,000 m)/0%	Shanghai (4 m)/0.59%	(Shi et al., 2020)
	2018	Tibet (4,000 m)/0%		(Ci et al., 2021)

Shanghai (4 m), Tibet (4,000 m), and Qinghai (3,000 m).

CRKP, carbapenem-resistant *Klebsiella pneumoniae*; CRAB, carbapenem-resistant *Acinetobacter baumannii*; CRPA, carbapenem-resistant *Pseudomonas aeruginosa*; MRSA, methicillin-resistant *Staphylococcus aureus*; VRE, vancomycin-resistant *Enterococcus*.

## Antimicrobial resistance in plateau areas worldwide

The Bolivian Altiplano lands are among the top 10 highlands in the world. They are located in the western part of South America, where the Andes Mountains, the longest mountain range in the world, are formed by the Cordillera mountain system running through North and South America. The Bolivian Altiplano has a total area of approximately 350,000 km^2^ and an average altitude of approximately 3,800 m. Straddling countries such as Peru and Bolivia, the Bolivian Altiplano covers an area of approximately 100,000 km^2^ in Bolivia, making Bolivia the highest-altitude country in the world. La Paz, the administrative capital of Bolivia, is located on a plateau at an altitude of >3,600 m, making it the highest-altitude capital in the world.

Bartoloni et al. (2016) studied the bacterial phenotype and molecular characteristics of tract infection at the Basico Villa Montes Hospital (altitude 1,875 m) in the Tarija region, Bolivia, and they found that the susceptibility rates of *E. coli* to amoxicillin–clavulanate potassium, cefotaxime, ceftazidime, meropenem, and sulfamethoxazole–trimethoprim were 56.5%, 89.4%, 92.3%, 100%, and 26.5%, respectively. The susceptibility rates of *K. pneumoniae* to amoxicillin–clavulanate potassium, cefotaxime, ceftazidime, meropenem, and sulfamethoxazole–trimethoprim were 47.3%, 68.4%, 73.7%, 100%, and 52.6%, respectively. In a study by Lopes et al. (2013) on the mechanism of the drug resistance of *AB* in the Cochabamba (altitude 2,548 m) area of Bolivia, 15 clinical isolates of *AB* were collected from three hospitals. Antimicrobial susceptibility tests showed that the minimum inhibitory concentration (MIC) value of two strains was >8 mg/L, which belonged to drug-resistant strains, and the drug resistance rate was 13.33%.

Bartoloni et al. (2006) compared the drug resistance of *E. coli* in 3,174 children in Bolivia and Peru. They found that the resistance rates in Camiri and Villa Montes hospitals in the Tarija region of Bolivia (altitude 1,875 m) to ampicillin, sulfamethoxazole–trimethoprim, tetracycline, streptomycin, chloramphenicol, nalidixic acid, kanamycin, gentamicin, and ciprofloxacin were 97%, 96%, 94%, 92%, 70%, 36%, 34%, 23, and 16%, respectively; the drug resistance rates in a Yurimaguas hospital in the Loreto region (altitude 104 m) and a Moyobamba hospital in the San Martin area (860 masl) of Peru to the aforementioned drugs were 92%, 91%, 91%, 79%, 71%, 38%, 22%, 20%, and 21%, respectively, among which there were significant differences in the resistance rates to ampicillin, sulfamethoxazole–trimethoprim, tetracycline, streptomycin, kanamycin, gentamicin, and ciprofloxacin between Bolivia and Peru. The prevalence of drug resistance in Bolivia was significantly higher at high altitudes than at low altitudes in Peru. Rodríguez et al. (2016) studied CRAB in South America and found that Bolivia (Cochabamba, altitude 2,548 m), Argentina (Buenos Aires, elevation 25 m; Mendoza, elevation 770 m), and Colonia (34 masl) had resistance rates >90%.

Sevillano et al. (2012) studied the prevalence and mechanism of drug resistance in *AB* in the Cochabamba region of Bolivia (altitude 2,548 m) and found that 35% of the isolates were resistant to imipenem. In addition, all isolates were resistant to aztreonam, cefazolin, cefoxitin, cefuroxime, fosfomycin, and nitrofurantoin, except for colistin, with the majority of the remaining antibiotics resistant to 75% of the isolates. In 2015, Versporten et al. (2018) analyzed the use and resistance to antimicrobial drugs in hospitalized patients in 53 countries worldwide and found that there were significant differences between regions, with the highest MRSA detection rate (10.4%) in Latin America and the lowest (1.2%) in Africa. The highest detection rate of *Enterobacter* ESBL in Eastern Europe was 37.7%, and the lowest in North America was 4.3%. The highest detection rate of GCephRE was 5.7% in Eastern Europe, and the lowest was 0.6% in Africa. Carbapenem-resistant Enterobacteriaceae had the highest prevalence (4%) in Latin America and the lowest in Northern Europe (0.2%). Carbapenem-resistant non-fermenting gram-negative bacilli were the highest in Eastern Europe (20.8%) and lowest in Western Europe (0.6%). The global average detection rates of MRSA and ESBL were 8.1%, the detection rate of GCephRE was 2.8%, and the detection rate of carbapenem-resistant Enterobacteriaceae was 1.2%; the detection rates of carbapenem-resistant non-fermented gram-negative bacilli were 5.3%, 8.1%, 2.8%, 1.2%, and 2.6%. Combined with the data from the China Antimicrobial Surveillance Network, the detection rates of MRSA, MRCNS, VRE, and GCephRE in the Qinghai–Tibet region were significantly different from those worldwide, and the detection rates of MRSA, MRCNS, and GCephRE were significantly higher than those in the global average and other regions.

We summarized and analyzed the detection rates of CRAB in the Qinghai–Tibet region of China, the Cochabamba region of Bolivia, and globally (**Table 2**). The detection rate of CRAB in the high-altitude areas of China was significantly different from that in the high-altitude areas of Bolivia; the detection rate in the Qinghai–Tibet region was lower than that in the Cochabamba region, whereas the global detection rate was between the rates of the Qinghai–Tibet region and the Cochabamba region. Many factors influence antimicrobial resistance, including health, national income, climate change, and antimicrobial use (Rahbe et al., 2023). In addition, owing to limited data, there is no comparison of drug resistance rates over time; therefore, further research is needed to address the reasons for the different detection rates of CRAB at high altitudes.

**Table 2:** Overview of CRAB in Qinghai–Tibet, Cochabamba, and Global.

Area	Year	Drug resistance rate (%)	References
Qinghai–Tibet	2014–2020	32.50%	(National Bacterial Drug Resistance Surveillance Network, 2021)
Cochabamba	2008–2009	35.00%	(Rahbe et al., 2023)
	2013–2014	90.00%	
Global	2016–2021	61.50%	(World Health Organization (2021)

Cochabamba (2,548 m).

CRAB, carbapenem-resistant *Acinetobacter baumannii*; Qinghai–Tibet, Qinghai and Tibet average drug resistance rate.

**Figure 2:**
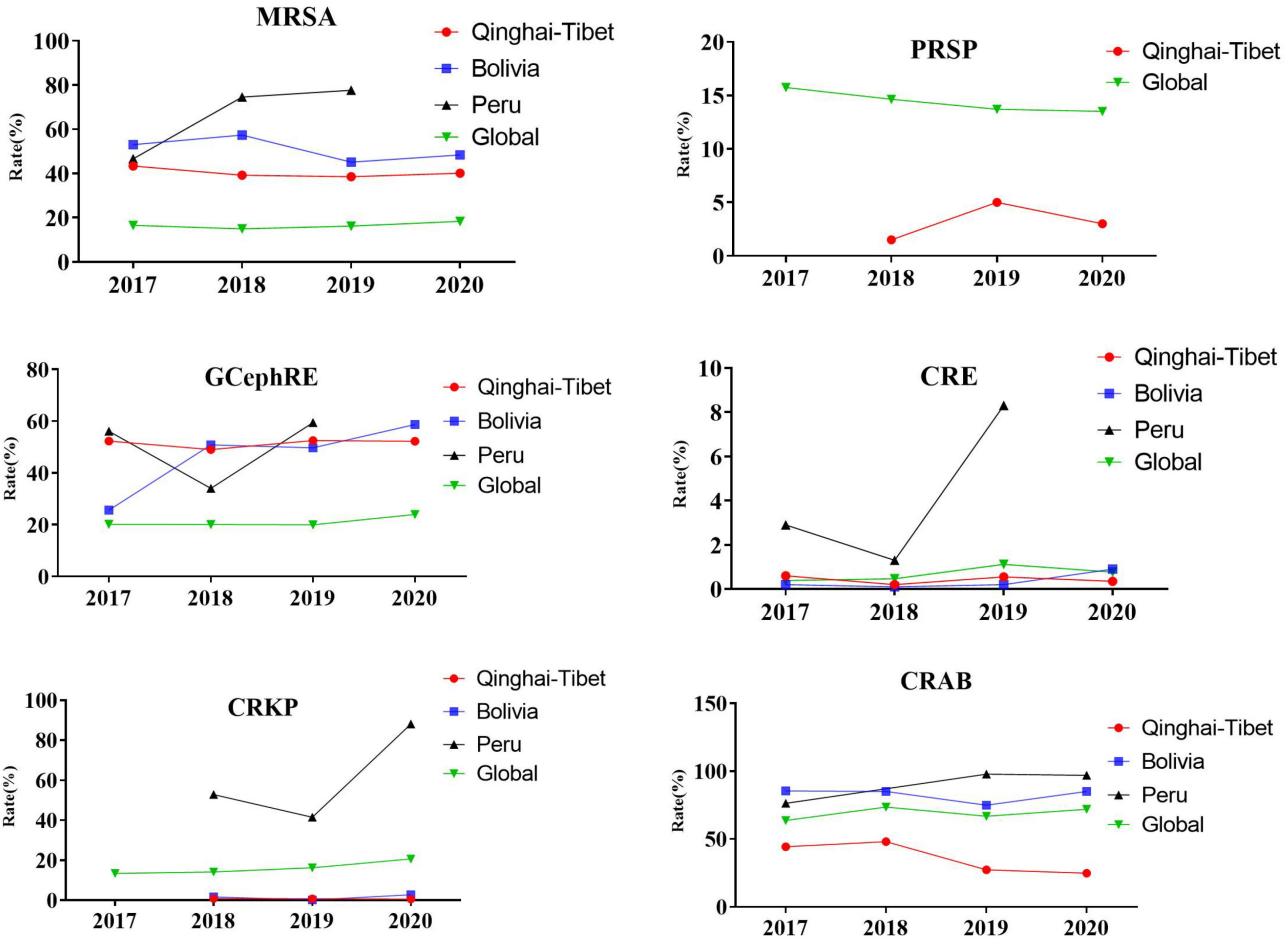
Prevalence of resistant organisms globally. From 2017 to 2020, detection rate of selected antimicrobial groups in Qinghai–Tibet, Bolivia, and Peru. MRSA, methicillin-resistant *Staphylococcus aureus*; PRSP, penicillin-resistant *Streptococcus pneumoniae*; GCephRE, third-generation cephalosporin Enterobacteriaceae; CRE, carbapenem-resistant Enterobacteriaceae; CRKP, carbapenem-resistant *Klebsiella pneumoniae*; CRAB, carbapenem-resistant *Acinetobacter baumannii*; Qinghai–Tibet, Qinghai and Tibet average drug resistance rate. Tibet (4,000 m), Qinghai (3,000 m), Bolivia (3,800 m), and Peru (1,555 m). Global drug resistance data from the World Health Organization (2017–2020).

Based on current literature and global drug resistance surveillance data, we analyzed the detection rates of MRSA, PRSP, GCephRE, carbapenem-resistant Enterobacteriaceae (CRE), CRKP, and CRAB between 2017 and 2020. Compared to the global detection rate, the trends of MRSA, PRSP, GCephRE, CRE, CRKP, and CRAB in the Tibetan Plateau, Bolivia, and Peru were significantly different. The detection rates of PRSP and CRAB were low in the Tibetan Plateau and continued to decrease for 19–20 years. The detection rates of PRSP, CRE, and CRKP were lower in the Tibetan Plateau and Bolivia, whereas the detection rates of MRSA and GCephRE were higher in Bolivia, the Tibetan Plateau, and Peru (**Figure 2**).

## Effects of hypoxia on bacterial resistance and related mechanisms

Currently, there are few studies on the effects of hypoxia on bacterial resistance, and only related studies have been conducted on *PA* (**Table 3**). Zang et al. (2014) discussed the correlation between hypoxia and the drug resistance of *PA* and found that a hypoxic environment increased the resistance of *PA* to ceftazidime, ciprofloxacin, levofloxacin, imipenem, meropenem, and aztreonam. They confirmed that the increased gene expression levels of mexE, mexA, and mexC in the efflux pump system were associated with drug resistance. Schaible et al. (2012) found that the resistance of *PA* to antimicrobial drugs increased under hypoxic conditions, and different degrees of hypoxia altered the expression of efflux pumps in *PA*, confirming that the increase in efflux pump expression was the relevant mechanism for the increase in the antimicrobial resistance of *PA*. In mammalian cells, a key signal in the hypoxia response is the inhibition of oxygen-dependent hydroxylases, leading to the activation of hypoxia-inducible factor (HIF)-1 and downstream genes. Schaible et al. found that HIF hydroxylase in *PA* is not involved in hypoxia to increase drug resistance, and other studies have reported that HIF hydroxylase is not the primary oxygen receptor in bacteria (Kaelin and Ratcliffe, 2008). In addition, Schaible et al. (2020) investigated the potential role of oxygen receptors [*Pseudomonas* prolyl hydroxylase (PPHD)] in controlling the virulence and antibiotic resistance of *PA*, and they found that *PA* lacking PPHD exhibited increased virulence associated with increased bacterial motility and that PPHD-deficient *PA* enhanced resistance to tetracycline-related antibiotics by increasing the expression levels of efflux pump transporters. Thus, PPHD is a potential bacterial oxygen receptor linking microambient oxygen levels to *PA* virulence and antibiotic resistance. Furthermore, Sherrard et al. (2014) found that cystic fibrosis in the lungs can increase antimicrobial resistance and may be associated with its hypoxic environment in cystic fibrosis.

**Table 3:** Sensitivity of *Pseudomonas aeruginosa* under hypoxia and related mechanisms.

Bacterial	Model	Changes in antibiotic resistance	Mechanism	References
PA	Hypoxia chamber (10%–12% oxygen) for 20 h	MIC of CAZ, CIP, LEV, IPM, MEM, and ATM: ↑	The mRNA expression of mexE, mexA, and mexC was increased	(Zang et al., 2014)
PA	Hypoxia chamber (1% oxygen) for 6–24 h	MIC of FOT, CAZ, CIP, TIM, and TZA: ↑	The mRNA expression of mexE, mexA, and mexC was increased	(Schaible et al., 2012)
PA	Hypoxia chamber (1% oxygen) for 18–24 h	MIC of tetracycline antibiotics (TC, DOX, MINO, and TGC): ↑	The mRNA expression of mexEF-OprN was increased. Efflux pump mexEF-OprN was upregulated in the absence of PPHD	(Schaible et al., 2020)

PA, *Pseudomonas aeruginosa*; CAZ, ceftazidime; CIP, cefepime; LEV, levofloxacin; IPM, imipenem; MEM, meropenem; ATM, aztreonam; FOT, cefotaxime; TIM, ticarcillin–clavulanic acid; TZA, piperacillin–tazobactam; TC, tetracycline; DOX, doxycycline; MINO, minocycline; TGC, tigecycline; MIC, minimum inhibitory concentration; PPHD, *Pseudomonas* prolyl hydroxylase.

## Conclusions

Based on data from the China Antimicrobial Surveillance Network and the study of microbial resistance in the plateau area, we compared microbial resistance rates in the Qinghai–Tibet and plain regions and found significant differences between highlands and plains. The detection rate of MRSA in the plateau area was higher than that in the plateau area, and the detection rates of VREF, CRAB, and CRKP were significantly lower than those in the plain area. Currently, there is no prospective clinical study comparing the microbial resistance rates in plateau and plain areas, and the aforementioned results are based on a retrospective comparison of the current drug resistance rates in plateau and plain areas, which cannot control the experimental conditions and are prone to various deviations and confounding factors that affect the reliability of the research results and have certain shortcomings. Therefore, large-sample randomized controlled trials in plateau and plain areas are of great significance for a comprehensive understanding of the effects of plateau environments on microbial resistance. Based on previous literature and the global drug resistance data released by the World Health Organization, we analyzed the differences in the detection rates of some drug-resistant bacteria in Qinghai–Tibet, Bolivia, and Peru and worldwide, and we found that compared to the global detection rates, the trends of MRSA, PRSP, GCephRE, CRE, CRKP, and CRAB in the Qinghai–Tibet Plateau, Bolivia, and Peru were significantly different. These are all high-altitude regions, and according to the current literature, the microbial resistance rates between them are also significantly different. We found that economic conditions, antimicrobial control intensity, and rational drug use rate all affected the generation of drug-resistant bacteria. Therefore, the difference in antimicrobial resistance rate in plateau environments needs to be further verified.

Some studies have confirmed that hypoxia changes the drug resistance of *PA*, and the related mechanisms have been discussed. In a hypoxic environment, the MIC values of various antimicrobial drugs against *PA* were significantly increased, and expression of mexE, mexA, and mexC in the efflux pump system of *PA* was an important mechanism for increasing the rate of bacterial resistance. The data from the China Antimicrobial Surveillance Network showed that there is no significant difference between the antimicrobial resistance rate of *PA* in the plateau area and that in the plain area. Therefore, we speculate that the antimicrobial resistance rate and efflux pump effects of the simulated hypoxic environment, pathological microscopic hypoxic environment, and plateau hypoxic environment on bacteria may be different. Previous studies have found that a high-altitude hypoxic environment can significantly affect the activity and expression of internal and external efflux pumps in rats and may lead to drug resistance (Duan et al., 2021, 2020; Liu et al., 2023). There are few studies on the effects of hypoxia on bacterial efflux pumps, and only some studies have been conducted on *PA*. The changes of other bacterial efflux pumps have not been reported in the literature, and the mechanisms of hypoxia on other resistance mechanisms, such as β-lactamase and membrane porin, need to be further elucidated.

Plateau environments are unique and characterized by low oxygen, strong ultraviolet radiation, and cold temperatures. Currently, there are few studies on the influence of low-oxygen environments on microbial resistance, and there is no relevant research on the mechanism of bacterial resistance affected by these environments. There are many plateau areas worldwide, and human activities in these environments are becoming increasingly frequent. Therefore, it is of great significance to study the impact of plateau environments on microbial resistance for the rational use of drugs and curbing bacterial resistance in these areas.
